# Comparison of 7-Year, Real-World Clinical Outcomes between Drug-Coated Balloon Angioplasty versus Drug-Eluting Stent Implantation in Patients with Drug-Eluting Stent In-Stent Restenosis

**DOI:** 10.3390/jcm12134246

**Published:** 2023-06-24

**Authors:** Minsu Kim, Albert Youngwoo Jang, Joonpyo Lee, Jeongduk Seo, Yong Hoon Shin, Pyung Chun Oh, Soon Yong Suh, Kyounghoon Lee, Woong Chol Kang, Seung-Hwan Han

**Affiliations:** Department of Cardiology, Gil Medical Center, Gachon University College of Medicine, Incheon 21565, Republic of Korea; msgene@gilhospital.com (M.K.); likemed@gilhospital.com (P.C.O.); kangwch@gilhospital.com (W.C.K.)

**Keywords:** in-stent restenosis, drug-eluting balloon, drug-eluting stent

## Abstract

There are no data available comparing the real-world, long-term clinical outcomes of drug-eluting balloon (DEB) angioplasty and drug-eluting stent (DES) implantation in DES in-stent restenosis (ISR) lesions. We aimed to compare the real-world, long-term data available between DEBs and DESs in DES-ISR lesions. We analyzed consecutive DES-ISR lesions (225 lesions from 205 patients; male: 66.3%; mean age: 62.4 years) treated with either DEB or DES. The primary endpoint was target lesion revascularization (TLR), and the primary safety endpoint was the lesion-oriented composite outcome (LOCO). The LOCO is composed of cardiac death, myocardial infarction, and target lesion thrombosis during follow-up. During the 7-year follow-up period, TLR did not differ significantly between the DEB (*n* = 108) and the DES groups (*n* = 117) (HR: 1.07; 95% CI: 0.59–1.93, *p* = 0.83). The LOCO was significantly lower in the DEB group compared to the DES group (HR: 0.40; 95% CI: 0.16–0.98, *p* = 0.04), which was mainly driven by the lower levels of myocardial infarction (HR: 0.24; 95% CI: 0.06–0.94, *p* = 0.04) and the absence of target lesion thrombosis in the DEB group (vs. DES group 6%, *p* = 0.02). Additionally, cardiac death was found to be similar between the DEB and DES groups (HR: 0.56; 95% CI: 0.18–1.75, *p* = 0.32). DEB angioplasty showed favorable safety with a similar efficacy to that of DES implantation in DES-ISR lesions during the long-term follow-up period.

## 1. Introduction

Drug-eluting stent (DES) implantation has been established for treating coronary artery disease and is known to reduce the restenosis rate compared to that of bare-metal stents [1]. However, despite the emergence of a newer generation of DES, DES failure remains a problem that needs to be solved. The cumulative rate of target lesion revascularization (TLR) after DES-implantation within 5 years has been reported as 7–10%. A recent trial reported that approximately 20% of patients experienced in-stent restenosis (ISR) during the long-term follow-up period [2,3,4,5].

When it comes to the treatment of DES-ISR lesions, the treatment strategy can vary depending on the characteristics of the lesion and the physician’s preference. However, long-term data is still lacking to definitively determine the efficacy and safety of the different treatment approaches. Although it is a study focused on de novo lesions, the PICCOLETO II study presented significant findings which indicated a notable reduction in the occurrence of major adverse cardiac events (MACEs) and target vessel thrombosis after a 3-year follow-up, when comparing DEB therapy to contemporary drug-eluting stents (DES) [6]. Other studies have reported that thinner strut stents have shown lower rates of target lesion failure (TLF), myocardial infarction (MI), and stent thrombosis (ST) in small vessel and bifurcation lesions (with a diameter of 2.5 mm and less) [6,7]. Additionally, when comparing DES implantation to DEBs, there may be situations where the treatment strategy for DES has been determined based on the characteristics of the lesion, leading to the preference for the overlapping stents rather than a single long stent. The choice between DEB and DES treatment can be influenced by the specific nature of the lesion. Recent meta-analysis findings on this topic have indicated that overlapping stents (OLS) have higher rates of cardiac mortality and target lesion revascularization (TLR) compared to single long stents (SLS) [8].

A recent guideline has indicated that DEB angioplasty and DES implantation are recommended for DES-ISR lesions (class I, level of evidence A) [9]. In terms of the comparisons in clinical outcomes between the DEB and DES intervention groups in DES-ISR lesions, a patient-pooled meta-analysis of randomized ISR studies comparing the DEB and DES in patients with recurrent DES-ISR lesions revealed a slight decrease in the safety endpoints (including all-cause death, myocardial infarction, and target lesion thrombosis) with DEB angioplasty during the 3-year follow-up period. However, there was a significantly higher rate observed in the efficacy endpoint (target lesion revascularization) [10]. Currently, there are no real-world, long-term data (spanning over 5 years) available that could elucidate this important issue. Consequently, we conducted a comparison of real-world, long-term data between DEB and DES interventions in DES-ISR lesions.

## 2. Materials and Methods

### 2.1. Study Design and Patient Population

This was a single-center, retrospective, observational study comprising consecutive patients with DES-ISR lesions treated with either DEB angioplasty or additional DES implantation between April 2005 and March 2017, respectively. Patients aged 18 years and above who had DES-ISR lesions treated with either DEB or DES were included. Patients with (1) failed DEB or DES, (2) debilitating cancer with a life expectancy of less than one year, (3) DES-ISR lesions at veins or arterial grafts, and (4) cardiogenic shock at the time of the procedure, were all excluded from the study. ISR was defined as stenosis greater than 50% of the angiographic diameter within the coronary stent or its edge (with 5 mm margins proximal and distal to the stent). Revascularization was considered in selected patients with at least one of the following: (1) acute coronary syndrome, (2) documented myocardial ischemia using non-invasive tests, (3) at least one artery with >70% stenosis and stable angina, and (4) abnormal results on the invasive physiologic study. The therapeutic strategy was assigned by an attending interventional cardiologist. All patients had been taking dual anti-platelet therapy (aspirin and P2Y12 inhibitor) for at least 12 months after their index DEB or DES treatment, if not contraindicated. This study was approved by the institutional review board of the Gachon University Gil Medical Center (GDIRB2022-060) and complied with the Declaration of Helsinki (6th revision). After the full IRB panel have made their determination of risk and the need for informed consent, we have obtained a waiver of informed consent on the Gachon Medical Center Institutional Review Board.

### 2.2. Definition of Study Endpoints

The primary efficacy endpoint was ischemia-driven TLR, while the primary safety endpoint was the lesion-oriented composite outcome (LOCO). The LOCO is composed of cardiac death, MI, and target lesion thrombosis during a 7-year follow-up period. The secondary safety endpoint was each outcome of the LOCO.

All clinical events were adjudicated by the consensus of two cardiologists. Ischemia-driven TLR was defined as repeat revascularizations using percutaneous coronary intervention (PCI) or coronary artery bypass grafting (CABG) in DES-ISR lesions treated with DEB or DES, when the percentage of diameter stenosis (DS) was greater than 50% and associated with ischemic signs/symptoms, or when the percentage of DS was greater than 70% with or without the presence of ischemic signs/symptoms. [10]. The cause of death was considered as cardiac unless there was definite evidence of a non-cardiac cause. Spontaneous MI was defined as types 1, 2, and 3 MI based on the fourth universal MI definition [10,11].

### 2.3. Statistical Analysis

Data were analyzed using IBM SPSS Statistics (SPSS Statistics for Windows, Version 23.0., IBM Corp., Armonk, NY, USA) and MedCalc Statistical Software (version 19.4, MedCalc Software Ltd., Ostend, Belgium). Continuous variables were expressed as mean ± standard deviation, and discrete data were presented as absolute values and frequencies (%). Continuous variables were compared using a t-test or Mann–Whitney U test, and categorical variables were compared using either the chi-square test or Fisher’s exact test, as appropriate. For the main analyses, we used the log-rank procedure and the Cox proportional hazards model. Event-free survival curves were generated using the Kaplan–Meier method for all endpoints in both groups. A two-sided *p* value of less than 0.05 was considered statistically significant.

## 3. Results

### 3.1. Baseline LOCO-Oriented Clinical and Angiographic Characteristics

In total, 225 DES-ISR lesions (205 patients) were included in this study. Among these, 108 lesions (100 patients) underwent DEB, and 117 lesions (105 patients) underwent DES, respectively. The mean clinical follow-up period was 62.8 ± 37.7 months. The baseline LOCO-oriented clinical and angiographic characteristics are presented in Table 1. The mean age of the population was 62.4 ± 11.7 years and 66.3% of them were male. Among the clinical characteristics, the presentation of acute coronary syndrome was found to be significantly higher, and the ejection fraction tended to be higher in the DEB group compared to the DES group (*p* = 0.03 and 0.08, respectively). However, other clinical characteristics were found to not differ significantly between the two groups. Meanwhile, the ISR pattern was found to be significantly different between the two groups (*p* = 0.02) due to significantly higher rates of total obstruction pattern and edge involvement observed in the DES group compared to the DEB group (*p* = 0.01 and 0.02, respectively). However, there were no significant differences observed in the target ISR vessel, previously implanted ISR stent types (taxol-based, limus-based, and 1st or 2nd generation), diameter, and lengths of the ISR stents between the two groups.

### 3.2. Procedural Characteristics

In the DEB group, a paclitaxel-eluting balloon was used (SeQuent Please balloon catheter, B. Braun Melsungen, Berlin, Germany), and the diameter of DEB had to be at least the diameter of the pre-dilatation balloon. For lesion preparation, pre-dilation was routinely performed (100%). The mean diameter of DEB was 2.8 ± 0.3 mm. In addition, the mean length of DEB was 22.0 ± 5.5 mm, and the inflation time was 41.5 ± 20.5 s, respectively (Table 2). In the DES group, the mean diameter was 3.0 ± 0.4 mm, and the mean length was 24.5 ± 9.3 mm, respectively. Among these, 33 (28.2%) ISR lesions were treated with the first-generation DES, and 84 (71.8%) were treated with the second-generation DES, respectively (Table 3).

### 3.3. Primary Efficacy Endpoint, TLR

**Figure 1:**
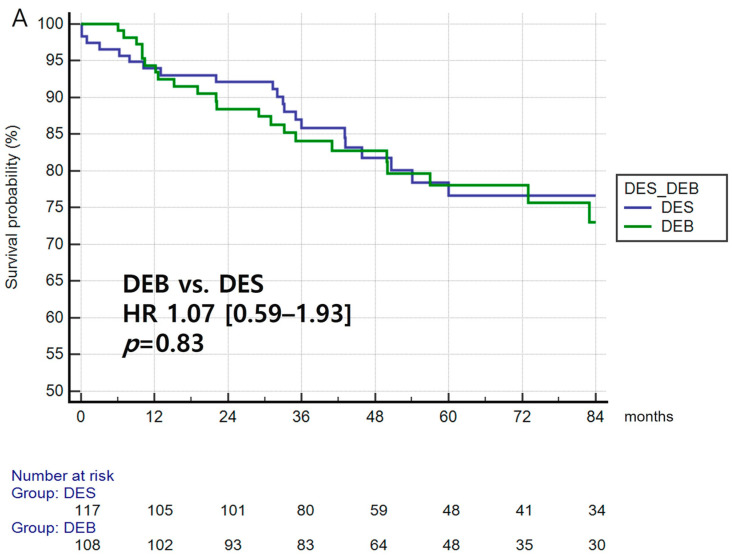

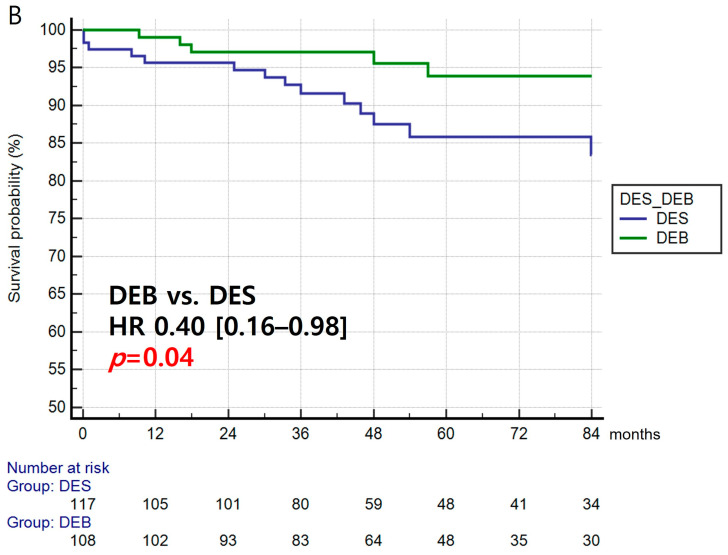
Primary efficacy endpoints. (**A**) Primary efficacy endpoint (target lesion revascularization)—cumulative incidence of the primary efficacy endpoint between the DEB and DES groups. (**B**) Primary safety endpoint (including cardiac death, myocardial infarction, and target lesion thrombosis)—cumulative incidence of the primary safety endpoint between the DEB and DES groups.

The primary efficacy endpoint, defined as TLR at 7 years, was found to have occurred in 44 patients (19.6%), and did not differ significantly between the DEB and DES groups (HR: 1.07; 95% CI: 0.59–1.93, *p* = 0.83) (Figure 1A). In addition, the TLR among the DEB, 1st generation DES, and 2nd generation DES groups were also found to non-significantly different (*p* = 0.71, Supplementary Figure S1A).

### 3.4. Primary Safety Endpoint

The primary safety endpoint (composed of cardiac death, MI, and target lesion thrombosis) was found to be significantly lower in the DEB group compared with the DES group (HR, 0.40; 95% CI [0.16–0.98], *p* = 0.04; Figure 1B). Although the primary safety endpoint in the DEB group did not differ significantly among the three subgroups (DEB, 1st generation DES, and 2nd generation DES) (*p* = 0.09), the DEB group showed a significantly lower rate of the primary safety endpoint compared with that of the 1st generation DES group (HR, 0.29; 95% CI [0.08–1.01], *p* = 0.03), Supplementary Figure S1B).

### 3.5. Secondary Endpoints

**Figure 2:**
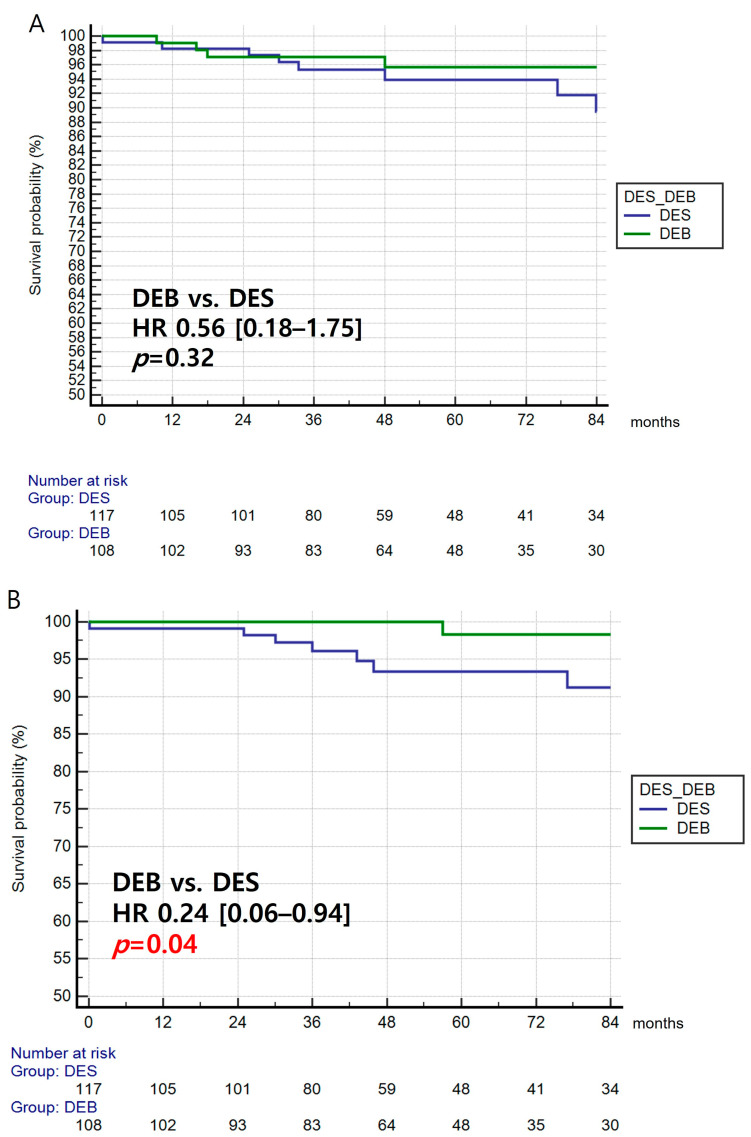

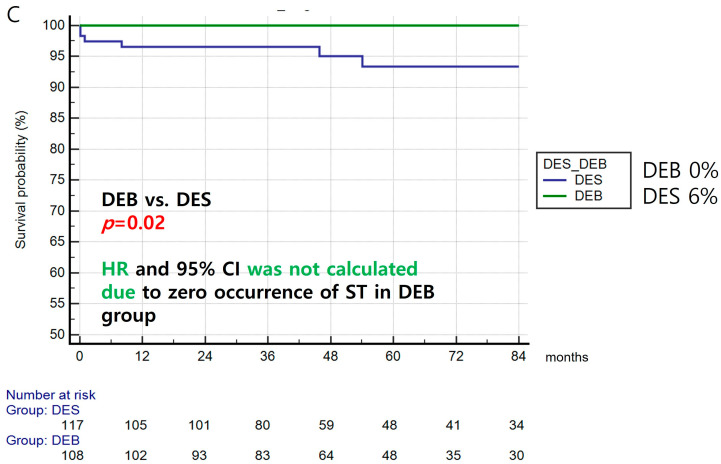
Secondary endpoints. (**A**) Cardiac death—cumulative incidence of cardiac death between the DEB and DES groups. (**B**) Myocardial infarction—cumulative incidence of myocardial infarction between the DEB and DES groups. (**C**) Target lesion thrombosis—cumulative incidence of target lesion thrombosis between the DEB and DES groups.

Cardiac deaths were not found to be significantly different between the two groups (HR, 0.56; 95% CI: 0.18–1.75, *p* = 0.32, Figure 2A) and among the three subgroup analyses (*p* = 0.60, Supplementary Figure S2A). Interestingly, MI was found to be significantly lower in the DEB group compared with that in the DES group (HR, 0.24; 95% CI: 0.06–0.94, *p* = 0.04, Figure 2B). The DEB group showed a significantly lower rate of MI than the 2nd generation DES group (HR, 0.14; 95% CI: 0.03–0.65, *p* = 0.03, Supplementary Figure S2B). Regarding target lesion thrombosis, there was no occurrence of target lesion thrombosis in the DEB group during the follow-up period. The risk of target-lesion thrombosis was determined to be significantly lower in the DEB group than in the DES group (*p* = 0.02, Figure 2C), and was consistent across the three subgroup analyses (*p* = 0.02, (Supplementary Figure S2C).

## 4. Discussion

To the best of our knowledge, since there are no real-world, long-term (spanning over 5 years) data on the clinical outcomes of DEBs and DESs in DES-ISR lesions, the current study is valuable in real-world clinical practice. The main findings of this study are as follows:

(1) The risk of the primary efficacy endpoint (TLR) was comparable between the DEB and DES groups;

(2) The DEB group showed a favorable primary safety endpoint composite of cardiac death, non-fatal MI, and target lesion thrombosis, mainly due to less MIs and the absence of target lesion thrombosis compared to the DES group;

(3) In the three subgroup (DEB, 1st generation DES, and 2nd generation DES) analyses, the TLR was determined to be similar among the three groups. DEB showed a lower rate of the primary safety endpoint compared to that observed in the 1st generation DES, and a lower rate of MI compared to that observed in the 2nd generation DES. In addition, the DEB group showed a lower rate of target lesion thrombosis compared to the 1st and 2nd-generation DES groups;

Although DES has drastically reduced the incidence of ISR, the treatment of DES-ISR lesions is particularly challenging compared to that with bare-metal stent-ISR lesions.

DEB angioplasty exhibits several advantages, such as the reduction of multiple metal layers used, saving large side branches, and avoiding prolonged dual anti-platelet therapy when compared with stent implantation. In a previous study, DEB angioplasty showed similar TLRs accompanied with a lower mortality and myocardial infarction rate compared to paclitaxel-eluting stent implantation during a 3-year follow-up [12]. In contrast, everolimus-eluting stent implantation in DES-ISR lesions showed a reduction in the occurrence of the major adverse cardiovascular events (MACE, including cardiac death, MI, and TLR) compared to that observed in DEB angioplasty during the 3-year follow-up period [13]. Therefore, there is still an ongoing debate regarding the efficacy and safety of DEBs and DESs in DES-ISR lesions. In a patient-pooled meta-analysis of ten previous randomized controlled trials comparing the outcomes of DEBs vs. DESs in ISR lesions, the DAEDALUS study showed that DEB angioplasty in DES-ISR lesions had a similar level of safety (including all-cause death, MI, and target lesion thrombosis) compared with DES implantation, but also displayed a higher rate of TLRs during the 3-year follow-up period [10,12]. Our current analysis is the first report on the clinical outcomes of DEBs vs. DESs in DES-ISR lesions in terms of long-term follow-up (7 years) and real-world clinical experience.

In the current study, DEB angioplasty was found to be safer. This is mainly due to the less myocardial infarctions observed along with the absence of target lesion thrombosis compared to the DES group and even in the three subgroups (DEB vs. 1st generation DES vs. 2nd generation DES) analyses. Interestingly, six cases of target lesion thrombosis occurred in the DES arm. However, no target lesion thrombosis was observed in the DEB group. The unfavorable safety of DES may be associated with the delayed endothelial healing and inflammation in the multiple layers of DESs, which are known risk factors for myocardial infarction and stent thrombosis [1,14]. This was supported by a meta-analysis demonstrating a lower level of MI with DEBs than with DESs [15]. In addition, aside from the stent-related and lesion-related factors, various elements related to medical therapy are also likely to have an impact. These factors include early dual anti-platelet therapy (DAPT) disruption, acute coronary syndrome (ACS) presentation, and high on-treatment platelet reactivity [16,17].

Recently, the ten-year outcomes of the ISAR-DESIRE 3 study, a prospective, open-label, randomized clinical trial comparing the treatment strategies of DEB vs. DES for DES-ISR lesions have been announced. In this study, over a ten-year follow-up period, there were no significant differences observed between the two groups in terms of the device-oriented composite outcome, which included cardiac death, target vessel myocardial infarction, target lesion thrombosis, and target lesion revascularization (multiplicity-adjusted log-rank: *p* = 0.610, Cox: HR 1.10, 95% CI: 0.79–1.52) [18]. However, there was an observed trend of increased death and cardiac death within the DES group during the 5-year period, suggesting a continued uncertainty regarding the long-term safety of DES implantation for DES-ISR treatment. This is consistent with our study, which showed a similar trend to the findings where DEB demonstrated comparable results to DES in terms of the primary efficacy endpoint, but displayed superior results compared to DES in the primary safety endpoint. In the ISAR-DESIRE 3 study, the authors concluded that further analysis is needed to assess the long-term safety of repeat DES implantations for DES-ISR. In the DAEDALUS study, although not statistically significant, there was a numerical trend observed indicating that DES showed a higher incidence of events in the primary safety endpoint, which includes all-cause death, myocardial infarction, and target lesion thrombosis, compared to DCB angioplasty in DES-ISR (8.7% vs. 7.5%, respectively; HR:1.13; 95% CI: 0.65 to 1.96) [10]. Therefore, we suggest that addressing the uncertainty regarding the long-term safety of DES can be achieved through a complementary analysis of real-world data and randomized controlled trials (RCTs). This approach can help address the concerns regarding the long-term safety of DES and provide more definitive answers.

This study had several limitations. First, this was a single-center, retrospective analysis. Due to its retrospective nature from a single center, this study was therefore prone to potential biases. However, a detailed long-term follow-up may be more accurate in a single-center study. Second, the sample size was relatively small. Third, the selection of the treatment strategy between the DEB and the DES was performed by attending interventionalists based on their knowledge and experience. Thus, the results of the current study may be influenced by their selection strategy. Fourth, the results of a few specific ISR patterns were not drawn in the current study. A more precise elucidation could have been achieved if comprehensive intravascular ultrasound (IVUS) data were available for each ISR lesion. However, due to the lack of IVUS assessments in all cases, it becomes challenging to pinpoint the exact contributing factors.

## 5. Conclusions

DEB angioplasty in DES-ISR lesions showed a more favorable safety with a similar efficacy to DES implantation during the long-term follow-up period.

## Figures and Tables

**Table 1:** Baseline lesion-oriented clinical and angiographic characteristics.

	DEB (*n* = 108)	DES (*n* = 117)	*p*-Value
Age, years	63.0 ± 11.1	62.2 ± 12.0	0.63
Male	69 (63.9)	79 (67.5)	0.58
HTN	66 (61.1)	67 (57.3)	0.59
Diabetes	50 (46.3)	48 (41.0)	0.50
Dyslipidaemia	57 (52.8)	65 (55.6)	0.69
Current smoking	21 (19.4)	16 (13.7)	0.28
ACS	97 (89.8)	92 (78.6)	0.03
EF	58.6 ± 10.4	55.9 ± 12.5	0.08
ISR location			0.44
LM	2 (1.9)	0	
LAD	55 (50.9)	73 (62.4)	
LCX	21 (19.4)	13 (11.1)	
RCA	30 (27.8)	31 (26.5)	
Previous DES stent:			
-Taxol-based stent	19 (17.6)	33 (28.2)	0.08
-Limus-based stent	89 (82.4)	84 (71.8)	
-1st generation DES	55 (50.9)	62 (53.0)	0.70
-2nd generation DES	53 (49.1)	55 (47.0)	
-Pre-stent diameter, mm	3.0 ± 0.4	3.0 ± 0.4	0.63
-Pre-stent length, mm	24.3 ± 6.6	25.6 ± 7.6	0.17
ISR pattern:			0.02
I (focal)	73 (67.6)	65 (55.6)	
II (diffuse intra-stent)	12 (11.1)	15 (12.8)	
III (proliferative)	13 (12.0)	11 (9.4)	
IV (occlusive)	10 (9.3)	26 (22.2)	
Total obstruction	10 (9.3)	26 (22.2)	0.01
Edge involvement	56 (51.9)	78 (66.7)	0.02
CTO	8 (7.4)	5 (4.3)	0.32

Values are mean ± SD or *n* (%) unless otherwise indicated. DEB, drug-eluting balloon; DES, drug-eluting stent; HTN, hypertension; ACS, acute coronary syndrome; EF, ejection fraction; ISR, in-stent restenosis; LM, left main; LAD, left anterior descending artery; LCX, left circumflex artery; RCA, right coronary artery; and CTO, chronic total occlusion.

**Table 2:** Procedural characteristics of the DEB.

Variable	
Pre-dilation, %	100%
DEB diameter, mm	2.8 ± 0.3
DEB length, mm	22.0 ± 5.5
Inflation time, s	41.5 ± 20.5
Inflation pressure, atm	10.1 ± 3.3

Values are mean ± SD or *n* (%) unless otherwise indicated. DEB, drug-eluting balloon.

**Table 3:** Procedural characteristics of the DES.

Variable	
DES diameter, mm	3.0 ± 0.4 mm
DES length, mm	24.5 ± 9.3 mm
Inflation time, s	17.1 ± 20.2 s
Inflation pressure, atm	13.2 ± 3.3 atm
1st generation stents	33 (28.2)
2nd generation stents	84 (71.8)

Values are mean ± SD or *n* (%) unless otherwise indicated. DES, drug-eluting stent.

## Data Availability

The datasets generated during and/or analyzed during the current study are available from the corresponding author on reasonable request.

## Supplementary Materials

The following supporting information can be downloaded at: https://www.mdpi.com/article/10.3390/jcm12134246/s1; Figure S1: Primary endpoints between between DEB, 1st generation DES, and 2nd generation DES groups. (A). Primary efficacy endpoint (target lesion revascularization). Cumulative incidence of primary efficacy endpoint between DEB, 1st generation DES, and 2nd generation DES groups. (B). Primary safety endpoint (including cardiac death, myocardial infarction, and target lesion thrombosis). Cumulative incidence of primary safety endpoint between DEB, 1st generation DES, and 2nd generation DES groups; Figure S2: Secondary endpoints between between DEB, 1st generation DES, and 2nd generation DES groups. (A). Cardiac death. Cumulative incidence of cardiac death between DEB, 1st generation DES, and 2nd generation DES groups. (B). Myocardial infarction. Cumulative incidence of myocardial infarction between DEB, 1st generation DES, and 2nd generation DES groups. (C). Target lesion thrombosis. Cumulative incidence of target lesion thrombosis between DEB, 1st generation DES, and 2nd generation DES groups.

## Abbreviations

CABG	Coronary Artery Bypass Grafting
DEB	drug-eluting balloon
DES	Drug-eluting stent
ISR	In-stent restenosis
TLR	Target lesion revascularization
LOCO	Lesion-oriented composite outcome
MI	Myocardial infarction
MACE	Major adverse cardiovascular event
PCI	Percutaneous coronary intervention
