# Supplementary material for: Comparison of 7-Year, Real-World Clinical Outcomes between Drug-Coated Balloon Angioplasty versus Drug-Eluting Stent Implantation in Patients with Drug-Eluting Stent In-Stent Restenosis
Source: J Clin Med. 2023 Jun 24;12(13):4246. doi: 10.3390/jcm12134246 (PMC10342484; doi:10.3390/jcm12134246)
Supplement: Supplementary file 1 [file jcm-12-04246-s001.zip › jcm-2436337-supplementary.pdf]

Supplementary Data

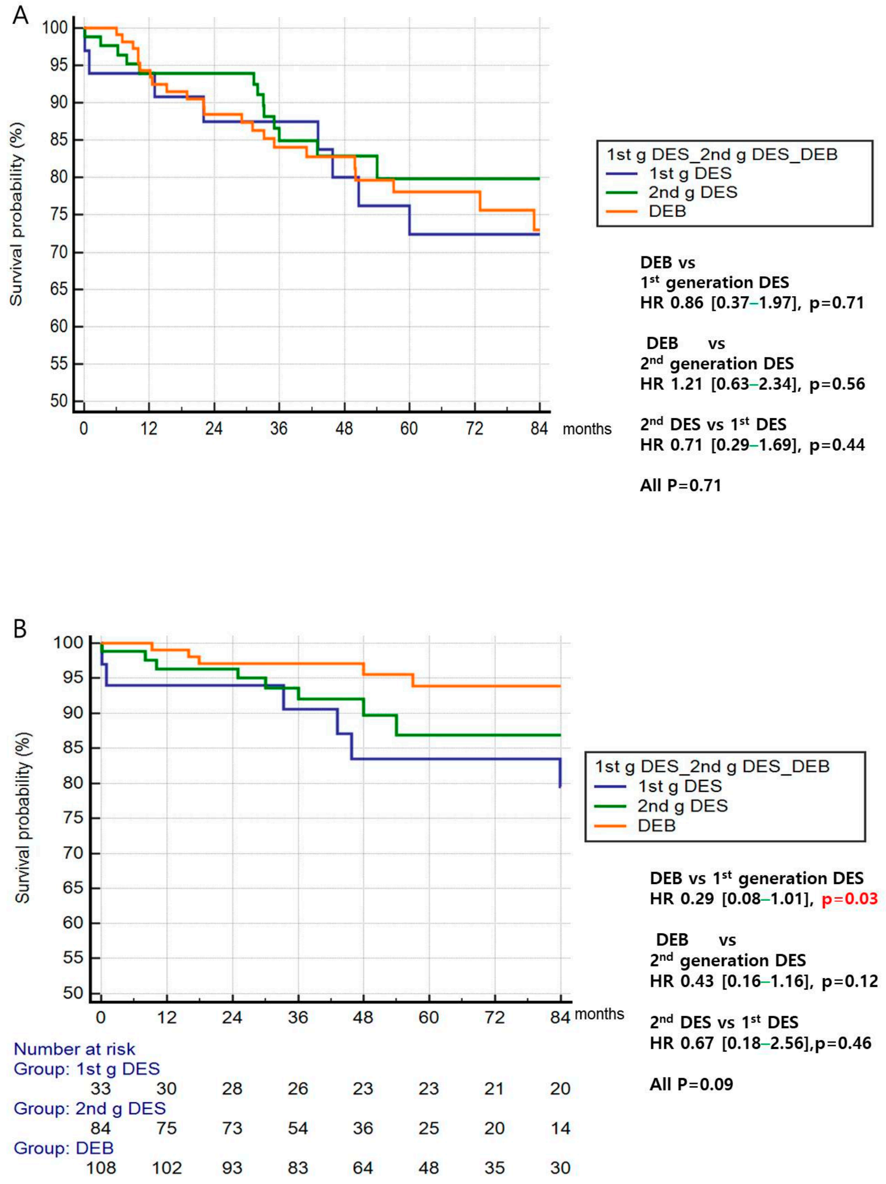

Figure S1. Primary endpoints between between DEB, 1st generation DES, and 2nd generation DES groups. (**A**). Primary efficacy endpoint (target lesion revascularization). Cumulative incidence of primary efficacy endpoint between DEB, 1st generation DES, and 2nd generation DES groups. (**B**). Primary safety endpoint (including cardiac death, myocardial infarction, and target lesion thrombosis). Cumulative incidence of primary safety endpoint between DEB, 1st generation DES, and 2nd generation DES groups.

A

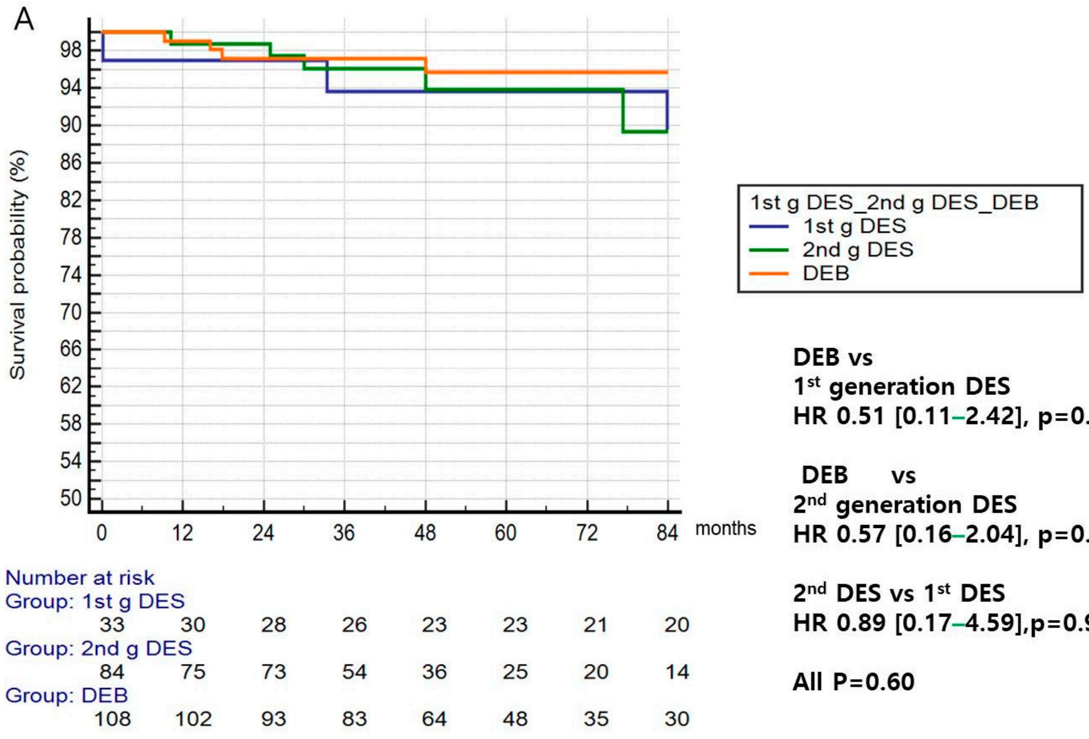

DEB vs
1st generation DES
HR 0.51 [0.11–2.42], p=0.33

DEB vs
2nd generation DES
HR 0.57 [0.16–2.04], p=0.40

2nd DES vs 1st DES
HR 0.89 [0.17–4.59],p=0.96

All P=0.60

B

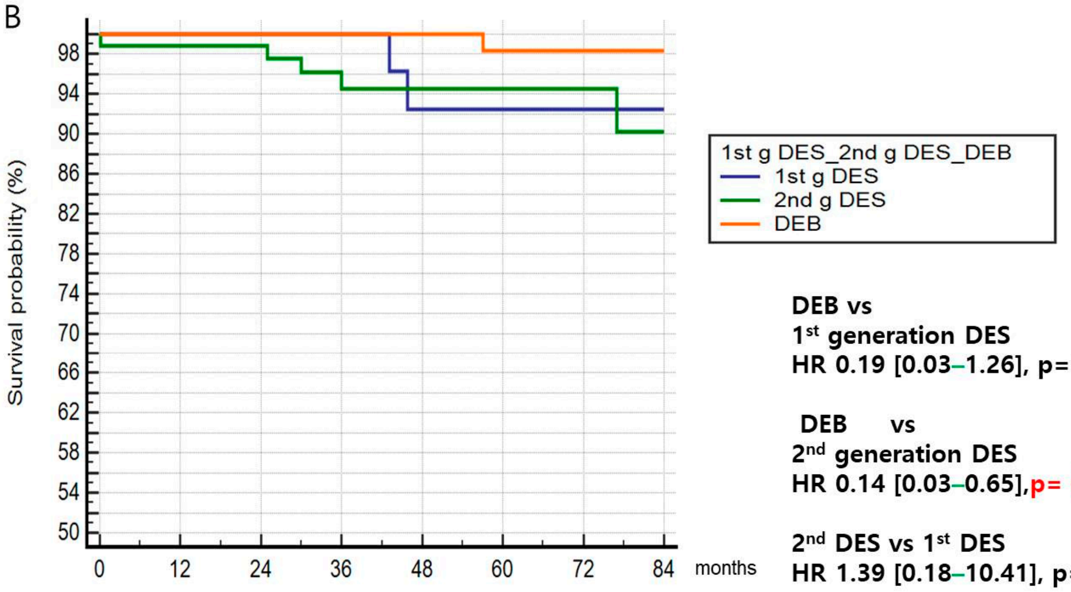

DEB vs
1st generation DES
HR 0.19 [0.03–1.26], p=0.14

DEB vs
2nd generation DES
HR 0.14 [0.03–0.65],p= 0.03

2nd DES vs 1st DES
HR 1.39 [0.18–10.41], p=0.75

All P=0.11

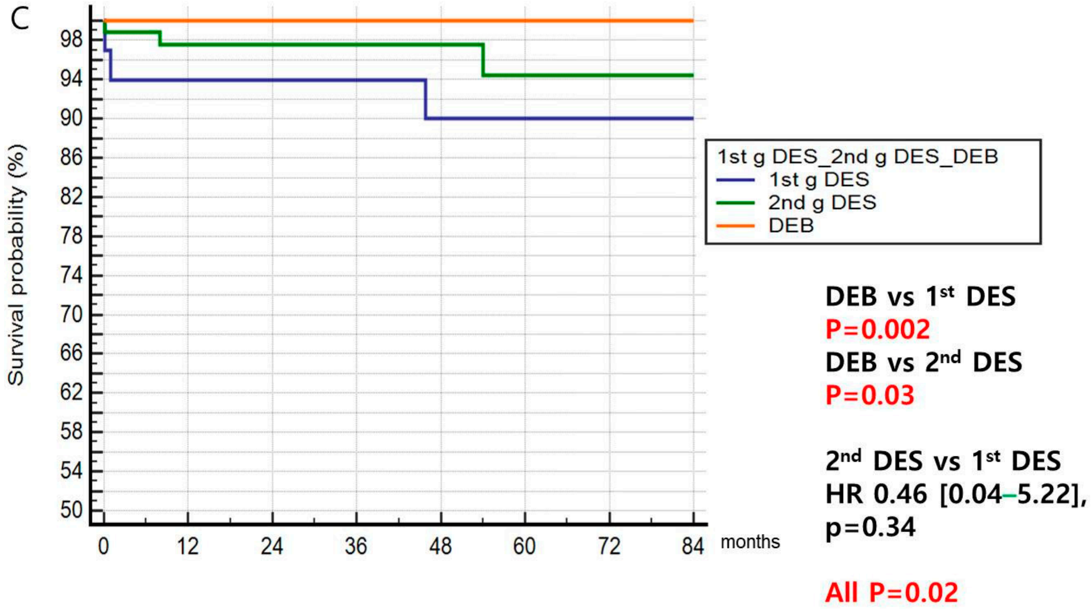

Figure S2. Secondary endpoints between between DEB, 1st generation DES, and 2nd generation DES groups. (**A**). Cardiac death. Cumulative incidence of cardiac death between DEB, 1st generation DES, and 2nd generation DES groups. (**B**). Myocardial infarction. Cumulative incidence of myocardial infarction between DEB, 1st generation DES, and 2nd generation DES groups. (**C**). Target lesion thrombosis. Cumulative incidence of target lesion thrombosis between DEB, 1st generation DES, and 2nd generation DES groups.
